# Evaluation of the estimate bias magnitude of the Rao’s quadratic diversity index

**DOI:** 10.7717/peerj.5211

**Published:** 2018-07-06

**Authors:** Youhua Chen, Yongbin Wu, Tsung-Jen Shen

**Affiliations:** 1 CAS Key Laboratory of Mountain Ecological Restoration and Bioresource Utilization & Ecological Restoration and Biodiversity Conservation Key Laboratory of Sichuan Province, Chengdu Institute of Biology, Chinese Academy of Sciences, Chengdu, China; 2 College of Forestry and Landscape Architecture, South China Agricultural University, Guangzhou, China; 3 Institute of Statistics & Department of Applied Mathematics, National Chung Hsing University, Taichung, Taiwan

**Keywords:** Biometrics, Forest ecology, Biodiversity measure, Estimation accuracy, Phylogenetic ecology, Functional traits

## Abstract

Rao’s quadratic diversity index is one of the most widely applied diversity indices in functional and phylogenetic ecology. The standard way of computing Rao’s quadratic diversity index for an ecological assemblage with a group of species with varying abundances is to sum the functional or phylogenetic distances between a pair of species in the assemblage, weighted by their relative abundances. Here, using both theoretically derived and observed empirical datasets, we show that this standard calculation routine in practical applications will statistically underestimate the true value, and the bias magnitude is derived accordingly. The underestimation will become worse when the studied ecological community contains more species or the pairwise species distance is large. For species abundance data measured using the number of individuals, we suggest calculating the unbiased Rao’s quadratic diversity index.

## Introduction

Biodiversity is constituted by multifaceted components. Measures of biodiversity thus should take into account species richness and abundance as well as other characteristics (like abundance evenness) quantified by information metrics, which are also valuable and should be incorporated. Rao’s quadratic diversity index is one of the most important biodiversity metrics that is widely applied to studies of functional and phylogenetic ecology (Rao, 1982, 2010; Mouchet et al., 2010). Its standard computation is to sum up the species’ distance between a pair of species *i* and *j* (*d_ij_*) that is weighted by the product of the relative abundances of both species (*p_i_* and *p_ij_*), given by $Q({\boldsymbol{p}}) = \sum\nolimits_{i \ne j} {{d_{ij}}{p_i}{p_j}} $ (Botta-Dukat, 2005; Ricotta, 2005a; Gusmao et al., 2016), where ${\boldsymbol{p}} = ({p_1},...,{p_S})$ represents the relative abundance distribution of the assemblage with *S* species. Here, species’ distance can be very flexible, ranging from phylogenetic to functional (or trait) distances (Ricotta, 2005b).

However, ecologists do not normally consider the statistical bias of Rao’s quadratic diversity index when applying it to practical research questions. Herein, statistical bias was used to measure the deviation of the expected estimate to the true value. Ecologists might think that the estimation bias issue of a species diversity index (including Rao’s quadratic diversity index investigated here) is not directly relevant to their own research, and the bias problem should instead be studied by statisticians. However, a key fact is that Rao’s quadratic diversity index is closely related to the Gini–Simpson index (Simpson, 1949; Magurran, 2004; Jost, 2006), which is well known and widely used by ecologists. For the Gini–Simpson index, it is commonly recognized that direct usage of the observed relative abundance of species (i.e., ${\hat p_i} = {X_i}/N$ known as the maximum likelihood estimate (MLE) of *p_i_*) will be statistically biased (particularly for small sample sizes which is usual in practical situations), and the biased-corrected estimator of the Gini–Simpson index is $Sim = 1-\sum\nolimits_i {{{{X_i}} \over N}\left({{{{X_i}-1} \over {N-1}}} \right)} $ (Simpson, 1949; Pielou, 1969; Hurlbert, 1971; Krebs, 1989; Magurran, 2004; Chen, 2015). To this end, evaluating the estimation bias of Rao’s quadratic diversity index is of great value for correctly applying it to research of trait-based functional or phylogenetic ecology (Pla, Casanoves & Di Rienzo, 2012; Swenson, 2014; Chen, 2015).

We do not claim that our study is the first one to study the estimation bias issue of Rao’s quadratic diversity index and propose an unbiased index because these have been well recognized by C.R. Rao himself and other researchers two decades ago (Nayak, 1983, 1986; Liu & Rao, 1995; Pons & Petit, 1996). However, we do believe our study can be valuable, as community ecologists rarely recognize the bias problem of the index or use an unbiased index in their practical research (Ricotta, 2004, 2005b; Hardy & Senterre, 2007). To this end, our study represents a recall on the application of the unbiased Rao’s quadratic diversity index (Nayak, 1983, 1986).

In summary, for the present paper, we explicitly derived the analytical bias magnitude of Rao’s quadratic diversity when the observed relative abundance of a species is used to directly compute the index. Using two empirical cases, we also demonstrate that the estimating bias of the routine calculation method can be very large. This calls for investigating the bias magnitude and removing the bias of the index. As a comparison, the bias and correction of the Gini–Simpson index are also demonstrated. The central goal of the study is helping ecologists to clearly understand why estimated biodiversity indices can be biased, and how large the amount of bias can be.

## Materials and Methods

### Estimate bias magnitude of the Rao’s quadratic diversity applied in phylogenetic or functional ecology

It is actually straightforward to prove that the routine computational method of Rao’s quadratic diversity index in community ecology is biased. To show this, we record the index as a function of the relative abundances of species as $Q({\boldsymbol{p}}) = \sum\nolimits_{i \ne j} {{d_{ij}}{p_i}{p_j}} $ for an ecological assemblage with a group of *S* species and a total of *N* individuals. As mentioned previously, ecologists use the observed species relative abundance (i.e., $\hat {\boldsymbol p} = ({\hat p_1},\ldots,{\hat p_S})$, where ${\hat p_i} = {X_i}/N$) when calculating the index (Ricotta, 2004, 2005a; Botta-Dukat, 2005; Gusmao et al., 2016); therefore, the observed Rao’s quadratic diversity index becomes

(1) $$Q(\hat {\boldsymbol p}) = \sum\limits_{i \ne j} {{d_{ij}}{{{X_i}} \over N}{{{X_j}} \over N}}, $$

which can also be recognized as the MLE of Rao’s quadratic diversity index.

We can expand this function using Taylor’s series at $\hat {\boldsymbol p} = \boldsymbol p$; thus, the resulting series is (Basharin, 1959):

(2) $${Q(\hat {\boldsymbol p}) = Q({\boldsymbol p}) + \mathop \sum \limits_{i = 1}^S {{\partial Q} \over {\partial {p_i}}}({{\hat p}_i} - {p_i}) + \mathop \sum \limits_{i = 1}^S \mathop \sum \limits_{j = 1}^S {{{\partial ^2}Q} \over {\partial {p_i}\partial {p_j}}}({{\hat p}_i} - {p_i})({{\hat p}_j} - {p_j}) + ...\;.}$$

After some algebraic manipulations by taking expectations on both sides of Eq. (2), we get

(3) $$E(Q(\hat {\boldsymbol p})) = Q({\boldsymbol p})-\sum\limits_{i = 1}^S {\sum\limits_{j = 1}^S {{d_{ij}}} {{{p_i}{p_j}} \over N}} .$$

A detailed derivation of Eq. (3) is given in Article S1. Equation (3) is one of our main conclusions: the standard calculation method of Rao’s quadratic diversity index $Q(\hat {\boldsymbol p})$ using observed relative species abundances (i.e., ${\hat p_i} = {X_i}/N$, for $i = 1,2,\ldots,S$) will statistically underestimate the true value of Rao’s quadratic diversity $Q(p)$. The magnitude of the underestimation is given by $\sum\nolimits_{i = 1}^S {\sum\nolimits_{j = 1}^S {{d_{ij}}} {{{p_i}{p_j}} \over N}} $. Interestingly, Eq. (3) also implies that there is a simple bias-correction formula for the quadratic diversity index, the derivation of which is presented in detail below.

### Bias correction of the Rao’s quadratic diversity

Using the common assumption that species abundances (quantified as the number of individuals) in an ecological community follow a multinomial distribution with rates (*p*_1_,…, *p_S_*) (Chao, 1981; Chao & Bunge, 2002; Shen, Chao & Lin, 2003; Chao & Jost, 2012; Chen & Shen, 2017; Shen, Chen & Chen, 2017) and the first and third equalities of Eq. S3 in Article S1, we have

(4) $$\eqalign{ Q({\boldsymbol p}) &= \sum\limits_{i \ne j} {{d_{ij}}{p_i}{p_j}} \cr  \quad \quad \;&{\rm{ = }} - {1 \over N}\sum\limits_{i \ne j} {{d_{ij}}{\rm Cov}({X_i},{X_j})} \cr  \quad \quad \; &= - {1 \over N}\sum\limits_{i \ne j} {{d_{ij}}\left\{ {E({X_i}{X_j}) - E({X_i})E({X_j})} \right\}} \cr  \quad \quad \; &= - {1 \over N}\sum\limits_{i \ne j} {{d_{ij}}E({X_i}{X_j}) + {1 \over N}} \sum\limits_{i \ne j} {{d_{ij}}E({X_i})E({X_j})} \cr  \quad \quad \; &= - {1 \over N}\sum\limits_{i \ne j} {{d_{ij}}E({X_i}{X_j}) + N} \sum\limits_{i \ne j} {{d_{ij}}{p_i}{p_j}} \cr  \quad \quad \; &= - {1 \over N}\sum\limits_{i \ne j} {{d_{ij}}E({X_i}{X_j}) + NQ{(\boldsymbol p)}}. \cr} $$

It should be noted that $E({X_i}) = N{p_i}$ was used here. Therefore,

(5) $$\matrix{{Q({\boldsymbol p})} {\, =\, \displaystyle{1 \over N}{1 \over {N - 1}}\mathop \sum \limits_{i \ne j} {d_{ij}}E({X_i}{X_j})} \hfill \cr {\quad\quad = \mathop \sum \limits_{i \ne j} {d_{ij}}E\left\{ \displaystyle{{{{X_i}} \over N}\left( {{{{X_j}} \over {N - 1}}} \right)} \right\}} \hfill. \cr } $$

By replacing the expectation operator with the observed counting of species’ individuals, the bias-corrected or unbiased Rao’s quadratic diversity index is given by

(6) $${\hat Q^U}({\boldsymbol p}) = \mathop \sum \limits_{i \ne j} {d_{ij}}{{{X_i}} \over N}\left( {{{{X_j}} \over {N - 1}}} \right).$$

Equation (6) also can be derived by using the fact that Rao’s quadratic diversity index is a weighted mean of all elements in the species pairwise distance matrix, derivation of which can be found in Article S2 (thanks Zoltán Botta-Dukát for providing the proof).

Therefore, the unbiased Rao’s quadratic diversity index, ${\hat Q^U}({\boldsymbol p})$, should be calculated using Eq. (6). Note that this unbiased form has been well known to statisticians (Nayak, 1983, 1986; Liu & Rao, 1995; Pons & Petit, 1996). The routine way of computing Rao’s quadratic diversity index using Eq. (1) in community ecology is statistically biased (as proven in Eq. (3)). Both Eqs. (1) and (6) look very similar; the only difference is the dominator in which one *N* has 1 subtracted from it for the unbiased formula. Apparently, for large sample sizes (*N* → ∞), Nayak (1986) theoretically proved that the standard estimator $Q(\hat {\boldsymbol p})$ is a consistent (or asymptotically unbiased) estimator of the true index, and thus both equations are nearly identical. However, when the sample size is very small, it is expected that the estimate bias of the true Rao’s quadratic diversity index using Eq. (1) will be very large. We will demonstrate this using two empirical tests along with a small example of a hypothetical assemblage for illustrating purpose in the following section. Moreover, we will demonstrate that the functional or phylogenetic distance, *d_ij_*, also greatly influences the bias magnitude. Note that, given *d_ij_* = 1 (when $i \ne j$) and *d_ij_* = 0 (when *i* = *j*), the unbiased Rao’s quadratic diversity index becomes the unbiased Gini–Simpson index, the derivation detail of which is given in Article S3.

### Numerical test and ecological applications

**Figure 1:**
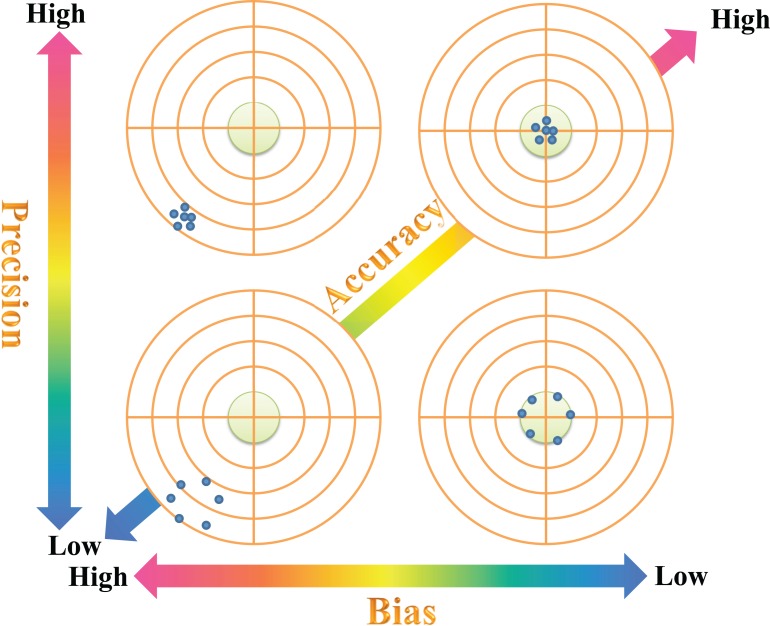
Measurements of bias, precision and accuracy in evaluating the performance of the bias-corrected Rao’s quadratic diversity index. The intersection of the vertical and horizontal lines represents the true value, while blue solid dots represent estimated values. Shaded circles in the middle of the targets represent high-accuracy zones when estimated values fall within them.

To show that the proposed bias-corrected index can accurately estimate the true value, we conducted one numerical test and two empirical tests. For each test, we quantified the bias magnitude along with the estimated accuracy of the proposed unbiased index compared to the original index (Fig. 1) (Bainbridge, 1985; Walther & Moore, 2005). Bias, measuring the deviation of the mean estimate to the true value, is an important component of estimation accuracy. Precision is another component of the estimation accuracy that measures the variance of the estimation. General relationships among estimating bias, precision and accuracy are given in Fig. 1.

In the numerical test, a hypothetical assemblage of three species (A, B and C) with relative abundances A = 1/6, B = 1/3 and C = 1/2 was employed to quick, numerically compare the biased and unbiased estimators in terms of bias, precision and accuracy, detailed meanings of which can one-to-one correspond to all measures of Fig. 1 but with numerical perspectives. For simplification, we set all phylogenetic distances = 1 among three species and fixed the sample size at *N* = 4. As a result, the true value of Rao’s quadratic diversity index can be specifically given by $Q({\boldsymbol p}) = {\rm{2}} \times {\rm{(1/6}} \times {\rm{1/3 + 1/6}} \,\times {\rm{1/2 + 1/3}} \times {\rm{1/2) = \,\,}}0.6111$, and there were four possible abundance patterns ignoring permutations of different species (see the first column in Table 1). The observing probability of each abundance pattern, which is calculated based on the joint probabilities of different possible permutations, is shown in the second column of Table 1. For example, observing the pattern (4, 0, 0), i.e., one species was present four individuals while the other two species were absent in the sample, had the probability of 0.0756. This value was calculated by summing the multinomial probabilities when the species present with four individuals was either A, B or C.

**Table 1:** Given a hypothetical assemblage of three species with relative abundances A = 1/6, B = 1/3 and C = 1/2, four abundance patterns along with the corresponding probabilities are demonstrated when four individuals were randomly sampled from the assemblage.

Abundance pattern	Probability	Estimator			
		${\hat Q^U}({\boldsymbol p})$	Empirical bias	$Q(\hat {\boldsymbol p})$	Empirical bias
(4, 0, 0)	0.076	0	−0.611	0	−0.611
(3, 1, 0)	0.364	0.5	−0.111	0.375	−0.236
(2, 2, 0)	0.227	0.667	0.056	0.5	−0.111
(2, 1, 1)	0.333	0.833	0.222	0.625	0.014

**Notes:**

For each abundance pattern, the bias magnitude of both estimators was calculated in detail for comparison. The overall statistical bias of the unbiased index is calculated as 0.076 * (−0.611) + 0.364 * (−0.111) + 0.227 * 0.056 + 0.333 * 0.222 = 0, and that of the biased index is computed as 0.076 * (−0.611) + 0.364 * (−0.236) + 0.227 * (−0.111) + 0.333 * 0.014 = −0.153. Moreover, the mean square error (MSE) of the unbiased and the biased indices are computed as 0.076 * (−0.611)^2^ + 0.364 * (−0.111)^2^ + 0.227 * 0.056^2^ + 0.333 * 0.222^2^ = 0.0499 and 0.076 * (−0.611)^2^ + 0.364 * (−0.236)^2^ + 0.227 * (−0.111)^2^ + 0.333 * 0.014^2^ = 0.0514. Though we used the root MSE (RMSE) for the two empirical cases, the difference between the RMSE and MSE is that the former is preserved to have the same unit as the estimator while the later is in a square scale of the RMSE.

In our empirical tests, the first dataset consists of biomass data of a plant community sampled from five plots in ultramafic soils of Tuscany, central Italy (Chiarucci et al., 1998; Ricotta, 2005b). In this dataset, because only the taxonomic classification of each species (subphylum: class: subclass: family: genus: species) is available, we assigned an equal weight (1/5) to each branch that connects a higher taxonomic unit (e.g., family) to a subsequent lower taxonomic unit (e.g., genus) (Ricotta, 2005b). The pairwise species distance, *d_ij_*, simply sums all of these equal weights from the most common taxonomic unit to each pair of species. Moreover, to make Rao’s index applicable, we assumed that a species’ relative abundance is proportional to the total biomass recorded for that species (herein the total biomass was summed as the recorded biomass in each plot). In another empirical dataset on the abundance of tree species on Barro Colorado Island (BCI) of central Panama (Condit, Hubbell & Foster, 1996; Condit et al., 2002; Volkov et al., 2003; Condit, Chisholm & Hubbell, 2012), the pair-wise species distance, *d_ij_*, was quantified using phylogenetic distances, for which a phylogenetic tree of 277 species was retrieved from the phylomatic database (http://phylodiversity.net/phylomatic/).

In our study, because the true value of Rao’s quadratic diversity index is insensitive to the sample size (i.e., the number of individuals of the local sample), we quantified the bias magnitude (BIAS) of the recommended and original Rao’s quadratic diversity indices when applied to estimate the true value of Rao’s quadratic diversity using local sampling data. Additionally, we also compared the overall estimated accuracy of the two indices with respect to the true value using the root mean squared error (RMSE). The estimated accuracy is the combination of both bias and precision (Walther & Moore, 2005). The general relationship between these quantities on measuring the estimation performance of a biodiversity index is presented in Fig. 1.

For revealing the bias magnitude on each estimator using different sample sizes, we considered seven cases: *N* = 30, 50, 100, 200, 1,000, 3,000 and 5,000. Note that the last three cases with large sizes are used to examine the asymptotical behavior of the standard estimator of Rao’s quadratic diversity index regarding its bias magnitude. Given a fixed sample size, we randomly sampled individuals from each of species abundance data with given relative abundances and distances *d_ij_*’s, and the sampling scheme was repeated 2,000 times for each scenario. As an illustration using the unbiased Rao’s quadratic diversity index (Eq. (6)), the explicit formulae of the two measures (BIAS and RMSE) are given as follows:

(7) $$\left\{ {\matrix{{{\rm{BIAS}} = \mathop \sum \limits_{i = 1}^{2,000} \displaystyle{{\hat Q_{(i)}^U({\boldsymbol p}) - Q({\boldsymbol p})} \over {2,000}}} \hfill \cr {{\rm{RMSE}} = \sqrt {{1 \over {2,000}}\mathop \sum \limits_{i = 1}^{2,000} {{\left( {\hat Q_{(i)}^U({\boldsymbol p}) - Q({\boldsymbol p})} \right)}^2}} } \hfill \cr } ,} \right.$$

where $\hat Q_{(i)}^U({\boldsymbol p})$ stands for the estimate of the proposed unbiased Rao’s quadratic diversity index using the simulated data from the *i*th replicate of the 2,000 replicates, and *Q*(***p***) represents the true value of Rao’s quadratic diversity. Phylogenetic distances between species are always fixed and consistently used over all simulation replicates.

## Results

For the numerical example, if we took a random sample of four individuals, the possible abundance patterns—(4, 0, 0), (3, 1, 0), (2, 2, 0) and (2, 1, 1)—will have respective probabilities of 0.0760, 0.3642, 0.2269 and 0.3333 to be observed in the sample (second column of Table 1). Among these abundance patterns, (3, 1, 0) possessed the highest likelihood (i.e., 0.3642) to be observed. As a consequence, if one had only a single data set in hand, the unbiased estimator could provide him with the highest probability to have a small empirical bias which was half smaller than that of $Q(\hat {\boldsymbol p})$. Although ${\hat Q^U}({\boldsymbol p})$ could lead to a larger empirical bias than $Q(\hat {\boldsymbol p})$ for the abundance pattern (2, 1, 1), the likelihood was small in comparison to all the other patterns (Table 1).

**Figure 2:**
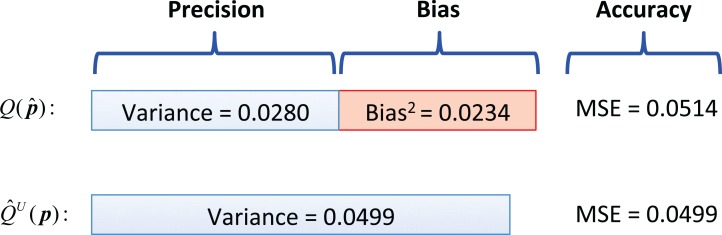
Comparison on the performance of the inbiased index against the biased index with the sample numerical example. The MSE (or equivalent to the RMSE) was illustrated as an accuracy measure by simultaneously taking the statistical bias and variance (in terms of precision) into consideration. For the biased index, Bias^2^ = (−0.153)^2^ = 0.0234; for the unbiased index, Bias^2^ = (0)^2^ = 0. Accordingly, the variance (reciprocal of precision) is the difference between MSE and Bias^2^.

To cope with the situation that the empirical biases of two estimators can vary with the selection of the sampling abundance patterns, we calculated the corresponding overall statistical bias induced by ${\hat Q^U}({\boldsymbol p})$ and $Q(\hat {\boldsymbol p})$ as zero and −0.153, respectively. This revealed that using the former was expected to have a much smaller bias than using the later. In addition to the statistical bias, the mean-squared-error (MSE; an effective measure when comparing the accuracy of different estimators) of ${\hat Q^U}({\boldsymbol p})$ and $Q(\hat {\boldsymbol p})$ were computed and given by 0.0499 and 0.0514, respectively. The MSE of an estimator can be categorized into two terms: the squared statistical bias and the variance of the estimator (Fig. 2), and one can see that the bias of $Q(\hat {\boldsymbol p})$ held a large proportion of its MSE, although both estimators had similar MSE values (Fig. 2).

Other than the above numerical example, as shown in Table 2, for both species abundance of the BCI forest plot and Italian plant communities, the original Rao’s quadratic diversity index always underestimated the true Rao’s quadratic diversity value. The underestimation magnitude (BIAS) became larger when the studied ecological community contained more species (by comparing the results of tree abundances in the BCI plot and plant communities surveyed in central Italy).

**Table 2:** Comparison of estimates of the true Rao’s quadratic diversity using biased and unbiased estimators on the two empirical datasets.

*N*	True ${\hat Q^U}({\boldsymbol p})$	MLE: $Q(\hat {\boldsymbol p})$			Unbiased: ${\hat Q^U}({\boldsymbol p})$		
		Avg	BIAS	RMSE	Avg	BIAS	RMSE
Plant community in Italy							
30	1.4480	1.3993	−0.0487	0.0727	1.4476	−0.0004	0.0558
50		1.4182	−0.0298	0.0480	1.4472	−0.0008	0.0384
100		1.4342	−0.0139	0.0301	1.4487	0.0006	0.0270
200		1.4408	−0.0072	0.0198	1.4481	0.0001	0.0185
1,000		1.4463	−0.0017	0.0078	1.4478	−0.0002	0.0077
3,000		1.4477	−0.0003	0.0046	1.4482	0.0001	0.0046
5,000		1.4478	−0.0002	0.0035	1.4481	0.0001	0.0035
BCI plot							
30	237.88	229.65	−8.23	16.74	237.57	−0.31	15.08
50		233.16	−4.71	12.20	237.92	0.05	11.48
100		235.86	−2.01	8.17	238.24	0.37	8.01
200		236.58	−1.29	5.76	237.77	−0.10	5.64
1,000		237.56	−0.32	2.55	237.80	−0.08	2.53
3,000		237.78	−0.09	1.51	237.86	−0.01	1.51
5,000		237.82	−0.05	1.11	237.87	−0.00	1.11

**Notes:**

Routine calculation method of the index and the bias-corrected method were computed using Eqs. (1) and (6), respectively. Avg denotes the average of estimates using 2,000 replicates, BIAS represents the magnitude of the bias, and the root mean squared error (RMSE) is used to reflect the estimate accuracy for each considered estimator.

In contrast, there were basically no differences between the estimated and true values when the unbiased Rao’s quadratic diversity index was used, as the bias magnitude BIAS was always close to zero as shown in the right panels of Table 2. The RMSE further demonstrated the estimated accuracy of the unbiased Rao’s quadratic diversity index in the estimation, which was always smaller when the unbiased estimator was calculated, regardless of the empirical datasets tested (Table 2).

When the sample size *N* became large, the bias magnitude of $Q(\hat {\boldsymbol p})$ was diminishing, and the RMSEs of the standard and unbiased estimators were almost the same as *N* ≥ 3,000, there was a tiny difference between them on the bias measure though (Table 2). As a consequence, our study on an empirical setting was in accordance with the theoretical derivation by Nayak (1986) that the standard formula $Q(\hat {\boldsymbol p})$ is an asymptotically unbiased estimator for the true Rao’s quadratic diversity index.

## Discussion

Development and testing of biodiversity indices are two of the most fundamental research components in biodiversity science and applied ecology. As mentioned already, the Gini–Simpson index (Simpson, 1949; Magurran, 2004; Jost, 2006) is one of the well-known diversity indices, the unbiased and biased formulas of which have been basic teaching materials in classical ecology textbooks (Pielou, 1969, 1977; Krebs, 1989; Magurran, 2004). Comparatively, as another important index, the Shannon index is well known to statistical ecologists to underestimate the true value when computed using observed species relative abundances (i.e., ${\hat p_i} = {X_i}/N$, for *i* = 1, 2,…*S*) (Basharin, 1959). However, so far, few ecologists have examined the statistical bias of some widely applied biodiversity indices, particularly from the sub-disciplines of phylogenetic and functional ecology. As mentioned earlier, Rao’s quadratic diversity index is one representative index in these sub-disciplines. Thus, our present work on the unbiased Rao’s quadratic diversity index call attention to, other than the unbiased Gini–Simpson index, which has become a part of classical textbook knowledge, the estimation accuracy of biodiversity indices.

It is nontrivial to recognize the issue of estimating bias for biodiversity indices, as the bias can greatly influence the accuracy, and further impact fair comparisons of biodiversity indices among ecological assemblages. This is easy to imagine, as an estimating bias will always exist for each of the different ecological assemblages and may be a nonlinear function of the community sizes of different assemblages (e.g., the bias term in Eq. (3) of Rao’s quadratic diversity index investigated here). To this end, adjustment or removal of the estimating bias of biodiversity indices has become critical and necessary in quantitative biodiversity research. In this study, we explicitly derived the bias magnitude when using the standard method to calculate Rao’s quadratic diversity index. The bias is related to both the sample size and phylogenetic distance of pairs of species (Eq. (3)), and the negative sign of the bias term implies that the original calculation routine of Rao’s quadratic diversity index will tend to underestimate the true value of the index an ecological assemblage is expected to have.

In summary, the present study emphasizes the importance of recognizing and correcting the statistical bias issue of diversity indices using Rao’s quadratic diversity index as a case study. We showed that the original calculation of the index using the observed species relative abundances would tend to underestimate the true value of the index. The bias magnitude was derived explicitly, and we showed that there was an analytical form for fully correcting the bias when the multiplier of the observed species relative abundance of a pair of species ${{{X_i}} \over N}{{{X_j}} \over N}$ is replaced by ${{{X_i}} \over N}{{{X_j}} \over {N-1}}$. Both the biased and unbiased indices looked similar, but in numerical tests, we showed that the bias of the original index (i.e., without a bias correction) tended to be more non-negligible for larger ecological communities or the distance between species was measured in divergence times (in units of million years ago) (e.g., the case study on BCI tree species as shown in Table 2). Conclusively, when applied to measuring functional and phylogenetic diversities in which the counting of species’ individuals is involved (Botta-Dukat, 2005; Ricotta, 2005b, 2005c), it is strongly recommended to use the unbiased Rao’s quadratic diversity index (Eq. (6)).

## Conclusions

The present study derived the bias magnitude of the Rao’s quadratic diversity index that is widely applied in functional and phylogenetic ecology. The bias magnitude $\sum\nolimits_{i = 1}^S {\sum\nolimits_{j = 1}^S {{d_{ij}}} {{{p_i}{p_j}} \over N}} $ is related to the community size, the pairwise species distances and their relative abundances. Accordingly, the unbiased Rao’s index is recommended for sampled species’ individual data, especially when large species pairwise distance *d_ij_* is involved. Moreover, by using a simple hypothetical example, we clearly demonstrate how to measure the estimation bias, variance (reciprocal of precision) and accuracy of a biodiversity index.

## Supplemental Information

10.7717/peerj.5211/supp-1 Supplemental Information 1 Supplemental Information. Detailed derivation on the estimation bias magnitude of Rao’s quadratic diversity index that is widely used in the ecological literature and the relationship between the unbiased Rao’s quadratic diversity index and the unbiased Simpson index.

10.7717/peerj.5211/supp-2 Supplemental Information 2 The two empirical datasets used for the present study. The first dataset contains the abundance and tree information of 277 tree species from BCI forest plot.
The second dataset contains the biomass and classification tree of 26 plants from the ultramafic soils of Tuscany, central Italy.

## Additional Information and Declarations

### Data Availability

The following information was supplied regarding data availability: The raw data are provided in the Supplemental File.
