# Supplementary material for: Evaluation of the estimate bias magnitude of the Rao’s quadratic diversity index
Source: PeerJ. 2018 Jul 6;6:e5211. doi: 10.7717/peerj.5211 (PMC6037161; doi:10.7717/peerj.5211)
Supplement: Supplemental Information 1 — Detailed derivation on the estimation bias magnitude of Rao’s quadratic diversity index that is widely used in the ecological literature and the relationship between the unbiased Rao’s quadratic diversity index and the unbiased Simpson index. [file peerj-06-5211-s001.docx]

**Supplemental Information**

*Supplemental Article S1:* *Detailed derivation on the estimation bias magnitude of Rao’s quadratic diversity index that is widely used in the ecological literature*

$\begin{matrix} Q(\hat{p})=Q(p)+\sum_{i=1}^{S} \frac{\partial Q}{\partial p_{i}}(\hat{p}_{i}-p_{i}) \\ \begin{matrix} & \end{matrix}+\sum_{i=1}^{S} \sum_{j=1}^{S} \frac{\partial^{2}Q}{\partial p_{i}\partial p_{j}}(\hat{p}_{i}-p_{i})(\hat{p}_{j}-p_{j})+... \end{matrix}$. (S1)

Recognizing that

$\left\{\begin{matrix} \frac{\partial Q(p)}{\partial p_{i}}=\sum_{i\neq j} d_{ij}p_{j}; \\ \frac{\partial^{2}Q(p)}{\partial p_{i}\partial p_{j}}=d_{ij}; \\ \frac{\partial^{k}Q(p)}{\partial p_{i}^{m}\partial p_{j}^{n}}=0\begin{matrix} . & m+n=k>2 \end{matrix} \end{matrix}\right.$. (S2)

Moreover, because it is well known that species abundance data are assumed to follow a multinomial distribution with rates $(p_{1},...,p_{S})$ (Chao, 1981; Chao & Bunge, 2002; Shen, Chao & Lin, 2003; Chao & Jost, 2012; Chen & Shen, 2017; Shen, Chen & Chen, 2017), we have the following quantities (Basharin, 1959):

$\left\{\begin{matrix} E(\hat{p}_{i})=p_{i} \\ E((\hat{p}_{i}-p_{i})^{2})=\frac{p_{i}(1-p_{i})}{N} \\ E((\hat{p}_{i}-p_{i})(\hat{p}_{j}-p_{j}))=-\frac{p_{i}p_{j}}{N} \end{matrix}\right.$. (S3)

Therefore, by taking expectations on both sides of Eq. 2 and using the facts in Eq. S3, we get:

$E(Q(\hat{p}))=Q(p)-\sum_{i=1}^{S} \sum_{j=1}^{S} d_{ij}\frac{p_{i}p_{j}}{N}$, (S4)

which is Eq. 3 in the main text.

*Supplemental Article S2:* *A simple derivation of the unbiased Rao’s quadratic diversity index*

We thank Dr. Zoltán Botta-Dukát for providing this simple but heuristic proof. Recalling that Rao's quadratic diversity index can be interpreted as mean distance between a pair of individuals randomly chosen from the sample, therefore, it can be calculated as,

,

where $w_{ij}=p_{i}p_{j}$ represents the probability that the first individual of the pair belongs to species *i* while the second one belongs to species *j*. Because $\sum_{i} \sum_{j} w_{ij}=1$, $Q(p)$ can be regarded as a weighted mean of all elements of the distance matrix, including zeros in the diagonal.

As mentioned above, since the first individual is expected to belong to species *i* while the second individual is expected to belong to another species *j*, it is easy to know that if $i\neq j$, the unbiased estimate of $w_{ij}$ is,

$w_{ij}\text{=}\frac{X_{i}}{N}\frac{X_{j}}{N-1}$.

By substituting this back to the previous formula, we get the unbiased estimator $\hat{Q}^{U}(p)$ for Rao's quadratic diversity index as shown in Eq. 6 of the main text.

*Supplemental Article S3:* *Relationship between the unbiased Rao’s quadratic diversity index and the unbiased Gini-Simpson index*

Note that, given $d_{ij}=1$, $i\neq j$; $d_{ij}=0$, otherwise, the unbiased Rao’s quadratic diversity index can include the unbiased Gini-Simpson index (*Sim*) as a special case from the derivation below:

$\begin{matrix} Q^{U}(p) = \sum_{i=1}^{S} \sum_{i\neq j} \frac{X_{i}}{N}\frac{X_{j}}{N-1} \\ = \sum_{i=1}^{S} \frac{X_{i}}{N}\frac{N-X_{i}}{N-1} \\ =\frac{N}{N-1}-\sum_{i=1}^{S} \frac{X^{2}_{i}}{N(N-1)} \\ =1+\frac{1}{N-1}-\sum_{i=1}^{S} \frac{X^{2}_{i}}{N(N-1)} \\ =1-\sum_{i=1}^{S} \frac{X_{i}(X_{i}-1)}{N(N-1)}=Sim \end{matrix}$.
